# Insomnia and pain in COVID-19 survivors: a cohort Tunisian study

**DOI:** 10.1192/j.eurpsy.2024.1041

**Published:** 2024-08-27

**Authors:** T. Mariem, B. Nadia, B. A. Houda, M. Islem, H. Najla, M. Sameh, K. Samy, E. Sahar, A. Jihen

**Affiliations:** ^1^Psychiatry “B”; ^2^Preventive medecine and hospital hygiene; ^3^Pneumology, Hedi Chaker university hospital, Sfax, Tunisia

## Abstract

**Introduction:**

The SARS-COV-2 infection emerging in 2019 caused over 600 million infected people worldwide leading to an explosion of multiple physical and mental health problems. In this study we brought the light to the persistent troubles in sleep and pain among the survivors of the pandemic.

**Objectives:**

We aimed to assess the prevalence of insomnia and the severity of pain among covid-19 survivors, and to seek an association between the two disorders.

**Methods:**

We conducted a prospective cohort study including 121 Tunisian COVID-19 inpatients who had been discharged alive from hospital. Each enrolled patient was asked about the period before the hospital stay, and the 6-9 month-period after hospital discharge, using *the visual analog scale* (*VAS*) to assess pain, *insomnia severity index (ISI) to evaluate insomnia severity and the mMRC (*modified British Medical Research Council*) to estimate dyspnea.*

**Results:**

The median age of participants was 59 years. Among them, 51.2% were females.

Our findings showed a significant increase in VAS score after COVID infection (1 [IQR (1-2] vs 3[1-6]; p<0.001) as well as with the ISI score (1 [IQR (1-1)] vs 5 [IQR (1-9)]; p<0.0001). The prevalence of insomnia and pain in long haulers was 30.56% and 26.4% respectively.

We found a significant correlation between insomnia and pain (p<0.0001, r=0.398). We also found a significant association between dyspnea and insomnia (p<0.0001) and between dyspnea and pain (p=0.001). The age of the patients was correlated with insomnia (p=0.028) and with dyspnea (p=0.007) but not with pain. Female gender was associated with developing insomnia (p<0.0001) and with pain (p=0.001) but not with dyspnea.

**Conclusions:**

Screening for persistent symptoms after the pandemic is important to help the survivors getting a better recovery in the long term.

**Disclosure of Interest:**

None Declared

